# Microarray analysis of circular RNAs in HCT-8 cells infected with *Cryptosporidium parvum*

**DOI:** 10.1186/s13071-021-04957-2

**Published:** 2021-09-21

**Authors:** Yuqing Wang, Heng Zhao, Yanan Zhang, Lei Yan

**Affiliations:** 1 School of Medicine, Cheeloo College of Medicine, Shandong University, Jinan, Shandong China; 2 Center for Reproductive Medicine, Reproductive Hospital Affiliated to Shandong University, Cheeloo College of Medicine, Shandong University, Jinan, Shandong China

**Keywords:** *Cryptosporidium parvum*, Statistics, Bioinformatics, Microarray

## Abstract

We read with great interest the article by Yin et al. (Parasit Vectors 14:238, 2021). The authors found that *Cryptosporidium* infection induced significantly aberrant expression of circular RNA profiles in HCT-8 cells, a finding which has far-reaching implications. However, due to the high number of false positives caused by multiple comparisons, statistical methods for microarray analysis should be carefully selected. Accurate analysis results will provide a convincing basis for subsequent experiments. In addition, we recommend several more appropriate methods in this article.

To the Editor,

With the development of high-throughput microarray and RNA sequencing technology, an increasing number of genes have been identified to be associated with parasitology [1, 2]. We read with great interest the article by Yin et al. [3]. These authors, using microarray, found that *Cryptosporidium* infection induced significantly aberrant expression of circular RNA (circRNA) profiles in HCT-8 cells. Their findings provide a fundamental basis to develop effective strategies against *cryptosporidiosis* and, consequently, have far-reaching implications.

In our opinion, it is necessary to further clarify the data analysis strategy of this study. Based on the authors’ description, they appear to use unadjusted *p*-values and fold change of expression values to define significantly differentially expressed circRNAs. However, due to the high number of false positives caused by multiple comparisons, statistical methods for microarray analysis should be carefully selected. Accurate analysis results will provide a convincing basis for subsequent experiments.

We suggest that the authors can adjust the *p*-values by the Benjamini–Hochberg correction to solve the problem of multiple comparisons, such as in the study of Atoyebi et al. [1]. Another option, as reported in a previous study, is linear modeling with empirical Bayes moderation, which provided good control of the false discovery rate as well as reasonable sensitivity when defining differentially expressed non-coding RNAs [4]. Therefore, we would like to suggest using Limma (linear models for microarray analysis) [5], which is an R/Bioconductor software package that uses linear models to analyze microarray and high-throughput PCR data [6]. Based on the output, the choice of the appropriate expression fold changes and false discovery rate < 0.05 as the cutoff is a conservative method to analyze changes in gene expression. A number of parasitological studies have used this statistical method and obtained satisfactory results [2]. We believe that this statistical method can provide a good technical support for parasitological studies.

We welcome further explanation of the data analysis strategy by the authors, which will make the results of this study more rigorous.

## Data Availability

Not applicable.

## Abbreviations

CircRNAs: Circular RNAs
Limma: Linear models for microarray analysis
